# The Physical History of Various Nations of the Earth with Special Reference to Their Teeth

**Published:** 1877-03

**Authors:** John Allen


### ARTICLE II.

### *The Physical History of Various Nations of the Earth with Special Reference to their Teeth.*

BY DR. J. ALLEN.

Having spent some thirty-eight years in dental practice, I have often been asked these two questions: first, "are not the teeth of the people of this country worse than those of other nations of the world?" And, second, "what is the cause of so many bad teeth in America?" These are two important questions involving the welfare of some thirty millions of inhabitants. In order to answer them satisfactorily, we have found it necessary to examine the physical history of mankind, in order to compare nations with nations in reference to their teeth, taking into consideration their food, habits, customs, climate, etc., etc.

In prosecuting these researches we find there are many nations whose teeth remain sound, even to old age, and it is as rare for them to lose a tooth as it is an eye or a limb. While in this country it is estimated that there are more than twenty millions of teeth lost annually from decay. And yet we find that the same general physical law which provides for the building up and sustaining the human structure, prevails among all nations, and that the divine architect of man has furnished an abundant supply of materials for all parts of the system. The body of man, with all its different parts and organs, is composed of only a few simple materials. These are combined in certain proportions, in order to give strength and utility to the whole structure. These materials are component parts of his food ; and although the nutrient substances used by the inhabitants of different parts of the world appear quite dissimilar, yet the food provided for them in various countries possesses the same general nutrient properties and chemical constituents everywhere that are essential for the human organism.

We will now proceed to notice some of the historical evidences which go to establish the fact that the Americans, as a whole, have worse teeth than the inhabitants of other nations. In portions of Europe, where the people, like the Americans, discard a large portion of the mineral element from their food, they also have bad teeth ; but among the Peasantry, and also in those sections where the inhabitants do not change the proportions of the mineral constituents of their food, they have good teeth.

But let us turn to the historical accounts of other countries where bolting cloths are not used for this purpose.

In Prichard's Researches into the Physical History of mankind he says: "The Albanians of Lesser Asia live principally on milk, cheese, eggs, olives and vegetables. Sometimes they bake bread, but often eat their corn or maize boiled." Hippocrates says they are very strong and muscular, have oral faces, a ruddy color in their cheeks, a brisk, animated eye, a well proportioned mouth, and *fine teeth.* In central Africa, north of the equator;—Prichard says : "The Mandingo tribes have the barbarous custom so common among the Pagans of Africa, of filling their teeth to a point."

In eastern Africa, among the different races of Abyssinians, we have the following description by this eminent author : "Their countenance is full without being puffed their eyes are beautiful, their mouth of moderate size, their lips thick, their *teeth white, regular, and scarcely projecting.*" Among the races of people inhabiting Nubia and other countries between Abyssinia and Egypt, Burckhardt says: "They are a handsome and bold people of a dark brown complexion, with beautiful eyes and *fine teeth.*" In the western parts of South Africa, comprising the Congo Empire, Proyart, who has graphically described it, says: "The negroes are well made, very black, with *white teeth* and pleasing countenances." In Dr. Oldfield's ethnographical researches in the interior of Africa, among the Felatahs, he says: "The color is light brown, features regularly formed, handsome mouth, thin lips, *with teeth as white as ivory.*"

We will now pass into Asia, and there among the mountain tribes of Dekham in India, Dr. Maxwell says: "The Khonds are a dark race of men, straight, well limbed, and free from obesity, which makes them have a tall appearance. Many of the men have a pleasing expression of the countenance. Generally, however, the nose is flattish, the cheek bones high, the face round, the lips and mouth large, displaying *fine teeth* The country produces rice, and most of the vegetables which are common in Europe." Among the Turkish tribes of Kiptschak, the Tartars of Kasan, says Erman, "Are of middle statue and muscular, but not fat. Their heads are of an oval shape, their countenances of fresh complexion, and fine, regular features; their eyes, mostly black, are small and lively; their noses arched, and thin, as well as their lips; their hair is generally dark, and their *teeth strong and white.*" We will now pass to that part of Asia between Hindostan and China, where we find, according to Finlayson, that the Siamese blacken their teeth, and redden their mouths with a masticatory of lime, catechu and betel, which gives them a disgusting appearance. Baron Larrey, who is well known as an eminent author on physical subjects, says: "The inhabitants of Eastern Arabia are somewhat above the average statue, robust and well formed. Their countenances oval, and copper colored, the forehead broad and elevated, the eyebrows black and bushy, the eye dark, deep seated and quick, the nose straight and of moderate size, the mouth well shaped, *the teeth beautiful and white as ivory.*" "In Egypt," the same author says, "the surface of the jaws of the Arabs are of great extent and in a straight or perpendicular line. The alveolar arches are of moderate size, and supplied with *very white and regular teeth*, the canines especially, project but little." The Arabs eat little and seldom of animal food.

We will now pass to a group of islands situated in the great Southern Ocean, between the eastern coast of Africa and the western shores of the new or American Continent. This group of islands received from Captain Cook, the name of the Society Islands. Mr. Ellis, who spent some six years among the inhabitants of Tahiti as a missionary, and had ample opportunity of observation, says: "These people are above the middle stature; in physical power they are inferior to the New Zealanders. The mouth of the Tahitian, he says, is well formed, though the lips are sometimes large, yet never so much so as to resemble those of the African. *The teeth are always entire* except in extreme old age, and though rather large in some, they *are remarkably white, and seldom either discolored or decayed.*"

Mr. Anderson, who visited New Zealand with Captain Cook, says: "The nations do not exceed the common stature of Europeans, and in general are not so well made, especially about the limbs. Their color is of a different cast, varying from a pretty deep black to a yellowish or orange tinge, and their features are also various, some resembling Europeans. Their faces are round, with full lips, their eyes large, hair black, straight and strong. *Their teeth are commonly broad, white and well set.*" Another writer, Captain Fitzroy, in describing the people of New Zealand, where he speaks of their teeth, says: "They are like those of the Tucgians, and, at the first glance, remind one of those of a horse. Either they are all worn down, in old persons, canine cutting teeth, and grinders, to an uniform height, so that their interior texture is quite exposed, or they are of a peculiar structure," undoubtedly the former. The natives who live near the hot, sulphurous waters on the borders of the lake of the Roturna, have the enamel of their teeth, especially their front teeth, yellow, although this does not impair their soundness, and is the effect, probably, of the corroding qualities of the thermal waters. To the eastward of the Society Islands, in the South Pacific, are the Gambier Islands. They are inhabited by a people fairer than the Sandwich Islanders. The average height of the men is about that of Englishmen, but they are not so robust. In their muscles their is a flabbiness, and in the old men a laxity of integument which allows their skin to hang in folds on different parts of their body. They have an Asiatic countenance, the teeth in the fourth class especially are not remarkable for evenness or whiteness, and seem to fall out at an early period. With reference to these physical characteristics, Dr. Prichard says: "Two causes may be assigned: the nature of their food and their indolent habits."

We will now pass to Easter Island, which is situated perhaps the most remote from the great continents of all inhabited islands on the globe. Captain Beechey has given the following physical account of the inhabitants. He says: "They are a fine race of people, especially the women. They have oval countenances, regular features, a high and smooth forehead, black eyes and *fine teeth.*"

Next, let us take a view of the San wan group of islands, situated also in the Pacific Ocean, in latitude thirteen and fourteen degrees. The inhabitants of these islands are strong, vigorous and well proportioned. Their features are all referable to a common type. This type is thus minutely described: "The nose is short and wide at the base, the eyes are black, and often large and bright, the forehead narrow and high, the mouth large and well filled with *white and strong teeth.*" These islands abound in pigs, dogs, fowls, birds and fish, and likewise in cocoa nuts, guava, banian trees and sugar canes. Belonging to another group in the same Ocean, are the Tarawan Islands. The people of this group differ from those above described. They are of middle size, their color is dark copper; their hair is fine, black and glossy, the nose slightly aquiline, the mouth is large, with full lips and *sound teeth.*

Vanikoro, another group of these islands, is also in this great Ocean. The sea coast is inhabited by a black race, who cultivate the taro, ignamus, bananas and the kava. "The inhabitants," says Dr. Urville, "belong to the black race of the great ocean approaching to that of proper negroes. They are generally small, their countenance has a singular resemblance to the ourang-outang, the eyes are large, and deeply set, resembling in form and color those of the negro. The lips are large, the chin small, the hair crisp. The use of the betel root destroys their teeth, and gives them a red tinge round the mouth. The women are horribly ugly, the old men are bald." Next we will proceed to the Archipelago, of the Fiji or Fejee Islands, which lie to the eastward of those above named, and are situated between fifteen and nineteen degrees of south latitude. This is a large group of islands, many of which are inhabited. The largest of this group is called the Great Viti. The people of this island are called Vitians. They are tall, well made, active and muscular. Their faces are broad, nose large and flat, large mouths, thick lips, and *sound, white teeth.*

The Fejeeans are generally above the middle height, and exhibit a great variety of figure. Their complexion is between that of the black and copper color races, although instances of both extremes are to be met with, thus indicating a descent from two different stocks. The faces of the greater number of the Fejeeans are long, with large mouth, *good and well set teeth.*

Leaving the Fejee Islands, we will pass to the coast of Australia. Captain Wilks, who was sent out by the

Government of the United States on an exploring expedition, says: "The natives of Australia differ from any other race of men in features, complexion, habits and language. Their color and features assimilate them to the African type, their long and black silky hair has a resemblance to the Malays; in their language they approximate more nearly to our American Indians, while there is much in their physical traits, manners and customs to which no analogy can be traced in any other people. Their color usually approaches chocolate, a deep umber, or reddish black, varying much in shade in different individuals. The cast of the face is between the African and Malay, the forehead usually high and narrow, the eyes small, black and deep set, the nose much depressed at the upper parts; the cheek bones are high, the mouth large and furnished with *strong*, *well set teeth*."

We will next direct your attention to the American races or tribes on this continent. Dr. Morton, who has published a very popular work on American skulls, says: "It is an old saying among travelers, that he who has seen one tribe of Indians, has seen all. So much do the individuals of this race resemble each other that notwithstanding their immense geographical distribution, and those differences of climate which embrace the extremes of heat and cold, there is a remarkable identity of physical characteristics throughout this whole race of people. All possess, alike, the long, lank, black hair, the brown or cinnamon colored skin, the heavy brow, the dull and sleepy eye, the full and compressed lips, and the salient but dilated nose."

Without following this author through his details of the physical characteristics of the American races, we will pass at once to his records of their teeth. He says: The cheek bones are large aud prominent, the upper jaw is often elongated, but the teeth are, for the most part vertical. The lower jaw is large and ponderous; *the teeth are also very large and seldom decay*, and few present marks of disease, though often worn by the mastication of hard substances."

With reference to the nations of the Western coast of North America, we have the following record from Captain Cook and Mr. Anderson:

"The visage of most of them is rather round and full, and sometimes also broad, with high, prominent cheeks. The nose flattened at the base, the forehead is rather low, the eyes small, black and languishing rather than sparkling, the mouth round, with thickish lips, *the teeth well set* but not remarkably white."

We will now direct attention to the nations of Chili, of California, and to those of the country near the Baie des Francais, who are of the Kolushian race. In the historical account of these people, by Mr. Rollin, we have the following: "They have rather a low forehead, black and lively eyes, nose of a regular shape and size, rather wide at the extremity; lips fleshy, a mouth of middle size, *fine and well set teeth.*" We will also notice the Peruvian nations. The physical characteristics of these nations in general are described by Dr. Orbigny. He says: "Their features have an entirely peculiar cast, which resembles no other American people but the Mexicans. Their head is oblong from the forehead to the occiput, somewhat compressed at the sides. The forehead is slightly arched, short, and falling a little back. Their face is generally broad, approaching to an oval form; their nose prominent, long, and strongly aquiline; the mouth is larger than common, though the lips are not very thick. The teeth are always *beautiful, even in old age.*" Dr. Orbigny says the mountaineers in South America are generally short, while the inhabitants of the plains are tall. "The Aroucans are a square, stout set of men with robust limbs, but without obesity; their joints large, their hands and feet small. Their heads are large in proportion to their body; the countenance full, round, with prominent cheek bones, large mouths, but thin lips. *Their teeth are good, and remain sound in old age.*"

"The aboriginal nations of Eastern Patagonia, says Captain Fitzroy, "Are a tall and extremely stout race of men. Their color is a rich, reddish brown. The head of the Patagonian is rather broad, but not high; the mouth is large and coarsely formed, with thick lips. Their teeth are usually *very good*, though rather large, and those in front have the peculiarity of being flattened, solid, and showing an inner substance." The following is an extract containing a description of the Pesherais, a people who inhabit one of the islands of the Magellanic Archipelago. (This extract is taken from an account of an exploring expedition sent out by the United States Government.)

"These people are not more than five feet high, of a light copper color. They have short faces, narrow foreheads, and high cheek bones. Their eyes are small and unusually black. Their nose is broad and flat, with wide-spread nostrils, mouth large, *teeth white, large and regular*." Dr. Orbigny in describing another tribe of the South American Indians, (called the Botocudos) says: "They wear for ornaments, collars or strings of human teeth." This is an evidence of their soundness and beauty. In the Northern division of South America we have the following physical description of a people called the Chaymas. Humboldt has given the following description of them:

"The countenances of the Chaymas, without being hard and stern, has something sedate and gloomy. The forehead is small and but slightly prominent. The eyes black, sunken, and very long. The wide mouth, with lips but little prominent, has often an expression of good nature. The nose and nostrils resemble those of the Caucasian race." "The Chaymas, says Humboldt, have fine, white teeth, like all people who lead a very simple life."

Having taken a cursory view of the physical charcteristics of several other nations of the world, with special reference to their teeth, we will now return to our own country. We have here a mixed population from various parts of the world, who have become so assimilated in habits, manners, customs, mode of living, etc., that the historian would recognize the same general characteristics of the people throughout the United States. But how different would be his record in reference to the teeth of the Americans at the present time from those nations herein referred to. He would tell you that very many of the people of this Country have narrow, contracted jaws, with crowded and badly decayed teeth. And in his statistics he would announce to you the startling fact that twenty millions of teeth are annually lost by the people of this Country. From the evidence which we have endeavored to bring before you, it will be seen that the teeth of the people of this Country are far worse than of any other here described. Mark the words of Humboldt when he said: "The Chaymas have fine teeth, like all people who lead a very simple life." It will be observed that in these historical researches, there is no evidence that the nations Humboldt alluded to attempted to improve their food by changing the proportions of the different constituents which the Creator has duly apportioned for the building up of organized beings. But, on the contrary, those nations use their food in the most simple forms, with all the constituents which nature placed there for the use of man. Another important fact in the history of those nations who have well developed jaws and teeth, should be also noted. It is this: they have plenty of exercise in the open air, which enables them to appropriate the different constituents in their food to the various parts and organs of the human system. From these different nations, therefore, we may learn some valuable lessons on the subject of the teeth. Although they have no Dentists nor Dental Literature, (for they need none) yet they learn much, as we may, from Nature, which will be found to tell exactly with true science.

Now let us turn again to our own records and see how widely we have departed from some of those physical laws which have been established by Omnipotence for our well-being. We have vainly attempted to improve our bread

(the staff of life) by changing the proportions of the mineral element in the flour we use, by bolting the most of it out and discarding it. Look for a moment at the gigantic scale upon which it is done in this country. According to our national statistics, of 1860, there were in in the United States 13,868 milling establishments for the manufacture of flour and meal, requiring 27,726 men, at an annual cost for labor of 8,721,391 dollars. Thus, you see, the number of men, mills, bolting cloths and dollars that are employed in this great *improvement* (?) devised by man for changing the proportions of one of the most important constituents in the staple article of food in this Country. The result of ignoring this mineral element from the staff of life is, undoubtedly, to a great extent one of the most prominent causes of this national calamity, that sweeps from the population more than 20,000,000 of teeth every year. The potter cannot make the bowl without the clay, neither can good teeth be formed without a due proportion of lime, which is abundantly provided for our use upon the outer portion of the grain, and in rejecting this portion of the cereals we virtually refuse to use the requisite materials of which the teeth are formed. We also deprive ourselves of a due proportion of atmospheric constituents, especially in our crowded cities. And also of the requisite amount of exercise to promote vigorous health and good constitutions. If we would be instrumental in doing more good in our profession, let us do all in our power to diffuse these important truths among the people.

---

